# Characteristics of Post-Exercise Lower Limb Muscle Tremor Among Speed Skaters

**DOI:** 10.3390/s25144301

**Published:** 2025-07-10

**Authors:** Szymon Kuliś, Przemysław Pietraszewski, Bianca Callegari

**Affiliations:** 1 Department of Rehabilitation, Józef Piłsudski University of Physical Education, 00-968 Warsaw, Poland; szymon.kulis@awf.edu.pl; 2 Institute of Sport Sciences, Academy of Physical Education, 40-065 Katowice, Poland; 3 Instituto de Ciências Exatas e Naturais, Universidade Federal do Pará, R. Augusto Corrêa 01, Belém 66093-020, PA, Brazil; callegaribi@gmail.com; 4 Laboratório de Estudos da Motricidade Humana, Universidade Federal do Pará, Av. Generalíssimo Deodoro 01, Belém 66073-000, PA, Brazil

**Keywords:** physiological tremor, speed skating, power spectrum density (PSD), neuromuscular adaptation, endurance training, sprint training

## Abstract

Physiological tremor analysis is a practical tool for assessing the neuromuscular impacts of sport-specific training. The purpose of this study was to examine and compare the physiological characteristics of lower limb resting postural tremor in athletes from Poland’s national speed skating team, following both sprint and endurance workouts. The study included 19 male, well-trained, elite athletes (with a mean age of 18 ± 3.1 years, body mass of 71.4 ± 10.1 kg, height of 178.5 ± 9.0 cm, and training experience of 12.6 ± 2.8 years) and a control group of 19 physically active but non-athlete men (with a mean age of 19 ± 2.3 years, body mass of 78.9 ± 12.1 kg, and height of 181.5 ± 11.0 cm). This group was assessed under resting conditions to provide baseline reference values for physiological tremor and to evaluate whether the neuromuscular tremor response is specific to trained athletes. Tremor amplitude and frequency were measured using an accelerometer, with data log-transformed to normalize the power spectrum distribution. Key findings indicate a significant effect of training condition on tremor amplitude in the low-frequency range (L(2_5); F_(1,18)_ = 38.42; *p* < 0.0001; η_p_^2^ = 0.68) and high-frequency range (L(9_14); F_(1,36)_ = 19.19; *p* < 0.0001; η_p_^2^ = 0.51). Post hoc analysis showed that tremor amplitude increased significantly after both sprint (*p* < 0.001) and endurance training (*p* < 0.001) compared to rest. No significant differences were observed between sprint and endurance training conditions for L(2_5) (*p* = 0.1014), but sprint training resulted in a greater increase in tremor in the high-frequency range (L(9_14); *p* < 0.0001). Tremor frequency (F(2_5) and F(9_14)) also increased significantly post-training. Notably, no differences were observed between limbs, indicating symmetrical neuromuscular adaptation. These findings highlight the utility of tremor analysis in monitoring neuromuscular fatigue and performance in speed skaters. Future research should explore the application of this method in broader athletic populations and evaluate its potential integration into training programs.

## 1. Introduction

The precise control and coordination of lower limb movements is essential for optimizing performance in a wide range of sports disciplines, including speed skating. Proficient lower limb control allows speed skaters to maintain stability, balance, and technique while navigating the ice at high velocities [1]. Speed skating places unique technical and physical demands on athletes, requiring exceptional lower limb strength, muscular endurance, and precise body positioning [2]. These demands make the physiological aspects of lower limb control particularly relevant in this sport. Muscle fatigue research includes various methods to detect this phenomenon. Muscle fatigue can be identified through several approaches, including electromyography (EMG) [3], mechanomyography (MMG) [4], the measurement of blood lactate levels [5], kinematic analysis using inertial measurement units (IMUs) [6] and near-infrared spectroscopy (NIRS) [7]. Blood lactate levels offer a general indicator of fatigue but are not suitable for real-time monitoring [8]. Wearable options include MMG, NIRS, and IMUs; however, NIRS and MMG face limitations such as time delays, with MMG being particularly prone to motion artifacts [9], while IMUs require further investigation into the link between kinematic metrics and muscle fatigue [10]. Surface electromyography (sEMG) is a commonly used wearable technique for real-time assessment of localized muscle fatigue by analyzing myoelectric signals [11]. Physiological tremor in the literature is defined as involuntary oscillations of body parts in healthy individuals, resulting from the interaction of mechanical and nervous factors [12,13]. Some authors, however, define it solely as a prevalent neurological disorder [14,15]. This type of tremor can be manifest as a symptom of fatigue and, in some cases, is considered by researchers to provide insight into training intensity and the athlete’s fitness level. However, the literature also suggests that the nature of physiological tremor resulting from physical activity remains insufficiently understood and studied. Speed skaters face significant and prolonged muscular demands during races, as their muscles are pushed to sustain performance under continuous and substantial load. This often results in neuromuscular fatigue, making them particularly susceptible to post-exercise muscle tremor [16]. This tremor can be observed as a symptom of fatigue, reflecting the strain placed on the muscles during the intense activity. Speed skaters must maintain a low, crouched position to minimize air resistance, which continuously stresses the muscles of the legs and core. Additionally, the specific posture required, combined with the high intensity and repetitive nature of skating strides, demands not only strength but also muscular control from the athletes. These factors collectively contribute to the appearance of post-exercise muscle tremor, and its potential impacts on recovery and stability [17]. Despite the importance of the precise control of lower limb muscles in speed skating, the physiological mechanisms responsible for muscle tremor after intense exercise have not yet been thoroughly investigated. In training, alternative measures to assess fatigue, rather than focusing specifically on tremor, often include exercise intensity monitoring through heart rate, energy expenditure during isometric contractions (quantified relative to the maximum voluntary isometric contraction, MVIC) [18], or muscle activation patterns via surface electromyography [19,20]. Research has shown that muscle tremor, which manifests as involuntary muscle oscillations following strenuous activity, can occur across different physical activities and training modalities [21,22]. The observed increases in tremor amplitude and shifts in frequency, particularly within the 9–14 Hz band following sprint training, may indicate central fatigue mechanisms. Central fatigue, as defined by Taylor et al. [21], involves a reduction in voluntary neural drive and altered cortical motor output. This interpretation is supported by Halliday et al. [23], who linked high-frequency tremor with motor unit synchronization disturbances. The persistence of tremor post-exercise further suggests that central mechanisms may outlast the rapid recovery of peripheral fatigue. These findings align with prior evidence that neuromuscular tremor can reflect cortical modulation and central nervous system strain under high-intensity training conditions. Studies particularly emphasize the relationship between muscle tremor and the physiological responses seen in endurance and strength-based activities [24]. Determining whether the frequency of muscle tremor varies between types of exercise could indicate that one training modality induces greater fatigue, thereby more significantly impacting movement control and neuromuscular stability. It seems reasonable that normal physiological postural tremor would have a direct impact on sports performance in disciplines requiring greater precision [25]. Some researchers suggest that the incremental increase in the amplitude of physiological tremor appears to depend more on the subjective perception of the effort’s magnitude than the actual work performed [26]. It is claimed that the mechanism of the increase in the amplitude of the tremor has its origin not, as could be assumed, in the muscles, but exclusively in the nervous system. Thus, changes in the amplitude of tremor reflect a state of fatigue manifested by a temporary disruption of control mechanisms in the nervous system. In the literature, physiological tremor in general is often associated with various neurological diseases [27]. Understanding the mechanical and neural components of tremor can help in designing training and enhancing sport performance, particularly in disciplines like speed skating [28]. Tremor analysis provides valuable insights into neuromuscular function, allowing for the identification of fatigue, asymmetries, and disruptions in motor control mechanisms that are critical for optimizing performance. By studying the underlying mechanisms and factors influencing tremor, targeted strategies can be developed to mitigate its effects, improve movement precision, and enhance performance consistency under demanding conditions [29]. Additionally, understanding tremor has broader implications, offering insights into neuromuscular function not only for athletic populations but also for individuals with neuromuscular disorders. Despite the growing interest in neuromuscular mechanisms in competitive sports, the specific physiological characteristics of lower limb tremor after intense exertion, such as sprinting or endurance training in speed skating, have not been sufficiently studied. Therefore, the purpose of this study was to examine and compare the physiological characteristics of lower limb resting postural tremor in athletes from Poland’s national speed skating team, following both sprint training and endurance workouts. 

The study proposed two hypotheses. The primary hypothesis is that the physiological characteristics of lower limb resting postural tremor will differ between sprint training and endurance workouts. Specifically, we hypothesize that sprint training, characterized by short bursts of high-intensity effort, will lead to greater neuromuscular fatigue and higher tremor amplitude in both low-frequency (L(2_5)) and high-frequency (L(9_14)) ranges, due to the intense activation of fast-twitch muscle fibers. Endurance training, which involves sustained moderate-intensity effort, will result in lower tremor amplitude but potentially more prolonged tremor activity, reflecting the cumulative fatigue of slow-twitch muscle fibers. The secondary hypothesis is that no significant differences in tremor characteristics will be observed between the right and left limbs, indicating symmetrical neuromuscular adaptation, regardless of training type or limb dominance.

## 2. Materials and Methods

### 2.1. Participants

This study was designed as a cross-sectional comparative investigation. Its primary aim was to examine postural lower limb tremor in elite speed skaters following different types of training (sprint vs. endurance) and compare the results to a non-athletic control group under resting conditions. Data collection occurred at single time points after each training modality, without repeated measures over time, random group allocation, or the experimental manipulation of independent variables. Therefore, the study is best characterized as a cross-sectional design with within-subject comparisons in the athlete group and between-group comparisons with the control cohort. This approach allowed us to explore training-specific neuromuscular responses while maintaining ecological validity in a high-performance athletic population. The study involved a group of 19 well-trained, elite male speed skaters (mean age: 18 ± 3.1 years; body mass: 71.4 ± 10.1 kg; body height: 178.5 ± 9.0 cm; and training experience: 12.6 ± 2.8 years), as well as a control group of 19 physically active but non-athlete men (mean age: 19 ± 2.3 years; body mass: 78.9 ± 12.1 kg; and body height: 181.5 ± 11.0 cm). None of the individuals in the control group had any history of competitive sports training or participation in organized sports programs. All control group participants were physically active (engaging in recreational activity 2–3 times per week), had no history of structured athletic or competitive sports training, and were instructed to refrain from intense physical activity for at least 48 h hours prior to testing. The study participants were Polish champions, medalists and finalists in the national youth speed skating championships. Athletes were in the pre-season phase of the season. All participants were healthy and reported no injuries in the past six months. Prior to the experiment, the participant underwent a 48 h rest period, during which they were instructed to maintain their regular diet and hydration. The tested athletes and non-athletes were informed about the purpose and methods of the research and about the possibility of withdrawing from the research at any stage. Legal guardians of the athletes gave their written consent to participate in the study in the case of minors. Prior to testing, each tested participant underwent a medical examination conducted by a nurse to rule out the presence of a cold, fever, or any condition that could serve as an exclusion criterion for the study. None of the participants had any neurological disease that could affect the tremor measurements. The research was conducted with the consent of the Senate Ethics Committee at the Józef Piłsudski University of Physical Education in Warsaw, Poland (SKE 01-31/2023). All procedures were carried out according to the Declaration of Helsinki.

### 2.2. Physiological Tremor Measurement of Lower Limbs

**Figure 1:**
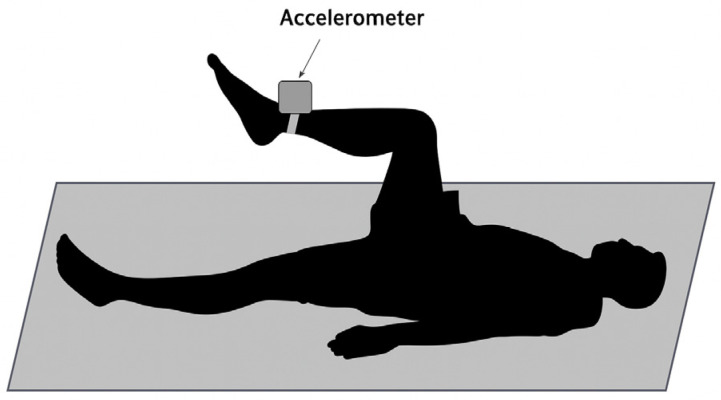
The placement of the accelerometer on the test subject and the testing position.

The testing procedure for muscle tremors of the lower limbs consisted of 3 measurements: at rest, after endurance training, and after sprint training. Each measurement was conducted with the athlete in a supine position on a hard surface, lying on a mat. The upper limbs were abducted to 90 degrees at the shoulder joints and externally rotated, with the dorsal side of the hand off the ground. The lower limb under examination was positioned in flexion to 90 degrees at both hip and knee joints, with the ankle joint placed in an intermediate position. An accelerometer (REJ 0006J manufactured by JBA ZB. Staniak, Warsaw, Poland) with a high signal-to-noise ratio, ensuring that only involuntary oscillations are recorded, was attached to a 1 kg weight placed 1 cm above the lateral ankle. According to a study by Tomczak [29], this inertial load, equivalent to less than 5% of the knee flexors’ maximum voluntary contraction (MVC), was designed to ensure it did not induce fatigue that could interfere with the physiological tremor measurement, even in potentially weaker participants, and enhances stability while ensuring that fatigue-induced tremor is not confounded by postural drift. The measurement duration for each limb was 34 s, during which the athlete was instructed to hold the tested limb as still as possible. The interval between testing the right and left limbs was 30 s. A 31.72 s waveform of the vertical acceleration component was recorded using the ZPP-3D/BC Acceleration Measurement Set (JBA Zb. Staniak, Poland) [30]. Figure 1 illustrates the placement of the accelerometer on the test subject and the testing position.

### 2.3. Procedures

Tremor measurements on the lower limbs of the speed skater group were conducted during a sports training camp for athletes. The initial measurement was performed at 11:00 a.m. on the first day, under resting conditions, with all participants well-rested and having abstained from any training activities during the preceding week and caffeine supplementation. Subsequent measurements were carried out following the endurance and sprint training sessions on the second and third days of the camp, respectively. These post-training measurements were conducted between 11:00 a.m. and 12:00 p.m., within ten minutes after completing each respective training session, as recommended by Gajewski [29]. The measurements of the non-athlete group served as a reference point for the physiological characteristics of tremor in individuals without systematic sports training. The non-athlete participants were examined only once, under resting conditions, in order to assess their baseline physiological tremor. The measurement took place in a sports hall, within the same time window as for the speed skaters, between 11:00 a.m. and 12:00 p.m. Data from both the right and left lower limbs were recorded, allowing for an analysis of inter-limb variability in tremor characteristics. The inclusion of the non-athlete group enabled a comparative evaluation to determine whether the physiological tremor of the lower limbs and its modulation by physical exertion are features specific to elite speed skaters.

#### 2.3.1. Sprint Training Session

The sprint training took place at the ice track and began with a warm-up consisting of a 10 min run, including dynamic and mobility exercises performed over a 10 m distance. These exercises included forward and backward arm circles, side steps, crossover steps, high knees (Skip A), butt kicks (Skip C), squat–jump combinations, forward and lateral leg swings, and forward bends with crossed legs. The warm-up concluded with static stretching targeting the quadriceps, hamstrings, adductors, and gastrocnemius muscles. The main training phase started with sprint drills performed over a 50 m distance in groups of five participants, each sprint repeated twice and with 30 s of recovery between efforts. Sprints progressed through three intensity levels (50%, 75%, and 100% effort) and included variations such as sprinting from a starting position, sprinting after holding a skating pose for 10 s, sprinting following five lateral crossover steps with a directional change, and sprinting triggered by a signal. Rest intervals of 3 min were maintained between sprint sets. 

Following sprint drills, athletes performed jump training. Each jumping exercise was repeated twice with 30 s intervals between repetitions. Exercises included five standard long jumps, five long jumps followed by acceleration, ten lateral skating-style jumps, and various box jumps (e.g., two-foot takeoff with two-foot or single-foot landings, single-foot takeoff with two-foot or single-foot landings, and sideways box jumps in a skating pose). Rest intervals of 3 to 5 min were maintained between jumping sets. The final component of the main phase involved imitation drills. These exercises were performed twice for each side, either for one minute stationary or over a 30 m distance. Static position exercises included holding a skating pose for two minutes and shifting the torso side-to-side with feet wide apart. Dynamic exercises mimicked skating movements, such as lateral hops, forward marching in a skating pose, and diagonal leaps forward. Resistance exercises were performed using a wide elastic band held by two participants, incorporating movements such as sideways walking (crossover step) in a skating stance, forward marching in the same stance, and sprinting from a starting position over 20 m. A rest interval of 10 min was taken before repeating the entire set. 

#### 2.3.2. Endurance Training Session

The endurance training session took place on a track and began with a 10 min warm-up phase, similar to the sprint session. Dynamic and mobility exercises were performed over a distance of 10 m, including forward and backward arm circles, side steps, crossover steps, high knees (Skip A), butt kicks (Skip C), squat–jump combinations, forward and lateral straight leg swings, and forward bends with crossed legs. The warm-up concluded with static stretching targeting the quadriceps, hamstrings, adductors, and gastrocnemius muscles. The main phase focused on sustained effort and cardiovascular endurance. It began with five laps of steady activity, on a 400 m track. This was followed by four sets of interval training, each set consisting of three repetitions of four minutes in a seated position alternated with one minute in a standing position. A five-minute recovery break was provided between each set. The main phase ended with five laps at a relaxed pace to promote active recovery. After the main phase, vibration measurements were conducted to assess potential muscle fatigue or physiological strain resulting from the exercise. The session concluded with a five-minute run at a gentle pace, followed by static stretching of the key muscle groups. This final phase was designed to support recovery, improve flexibility, and minimize post-exercise soreness. Because neuromuscular fatigue is typically measured during an isometric contraction following the sprint(s), an important implication is the time delay between the cessation of the sprint and the performance of the isometric contraction. It should be noted that this delay may be considered as a “recovery time” and can potentially lead to an underestimate in the development of peripheral or central fatigue following the sprint(s) because both peripheral and central fatigue can recover in approximately 2 min [31,32]. 

#### 2.3.3. Frequency Analysis

The acceleration signal was sampled at a frequency of 200 Hz and underwent low-pass filtering at the Nyquist frequency (100 Hz) prior to analog-to-digital conversion to mitigate the ‘mirror effect.’ The filtered signal was then subjected to frequency analysis to calculate the power spectral density (PSD) function, which characterizes the distribution of signal variance across the frequency spectrum. The dataset, consisting of 6144 samples, was segmented into six portions of 1024 samples each. The PSD for each segment was estimated using the fast Fourier transform (FFT) algorithm implemented in TDA1v0 software (version 1.0) developed and manufactured by Zbigniew Staniak JBA, Poland. A cosine window was applied during the analysis. The overall PSD function for the recorded signal was obtained by averaging the PSDs from all six segments. From the PSD function, indices describing the power and frequency of the tremor signal were calculated for each registered waveform. The distribution of power and amplitude in tremor signals is often represented using statistical models such as the log-normal distribution. Following the recommendation of Gajewski [33], a logarithmic transformation was applied to all variables representing the amplitude and power of the tremor signal, defined as the vertical-axis acceleration waveform. Log transformation normalizes the power spectrum, improving the interpretability and comparability of frequency-domain features. This transformation helps reduce the skewness of the data and enhances the sensitivity of statistical tests, which is especially beneficial when dealing with physiological tremor data with high variability across individuals. This transformation facilitates the quantification and analysis of tremors, providing insights into their behavior under various conditions. From the PSD function, indices describing the power and frequency of the tremor signal were calculated for each registered waveform.

The log amplitude indicator is defined as an average of logarithms of power components from the f_1_ to f_2_ frequency range:

(1) $$L\left(f_{1},f_{2}\right)=\frac{1}{f_{2}-f_{1}}\int_{f_{1}}^{f_{2}}lnPSD\left(f\right)df,$$

The mean frequency of power components from the f_1_ to f_2_ range defined as follows:

(2) $$F\left(f_{1},f_{2}\right)=\frac{\int_{f_{1}}^{f_{2}}f\cdot PSD\left(f\right)df}{\int_{f_{1}}^{f_{2}}PSD\left(f\right)df}.$$

**Figure 2:**
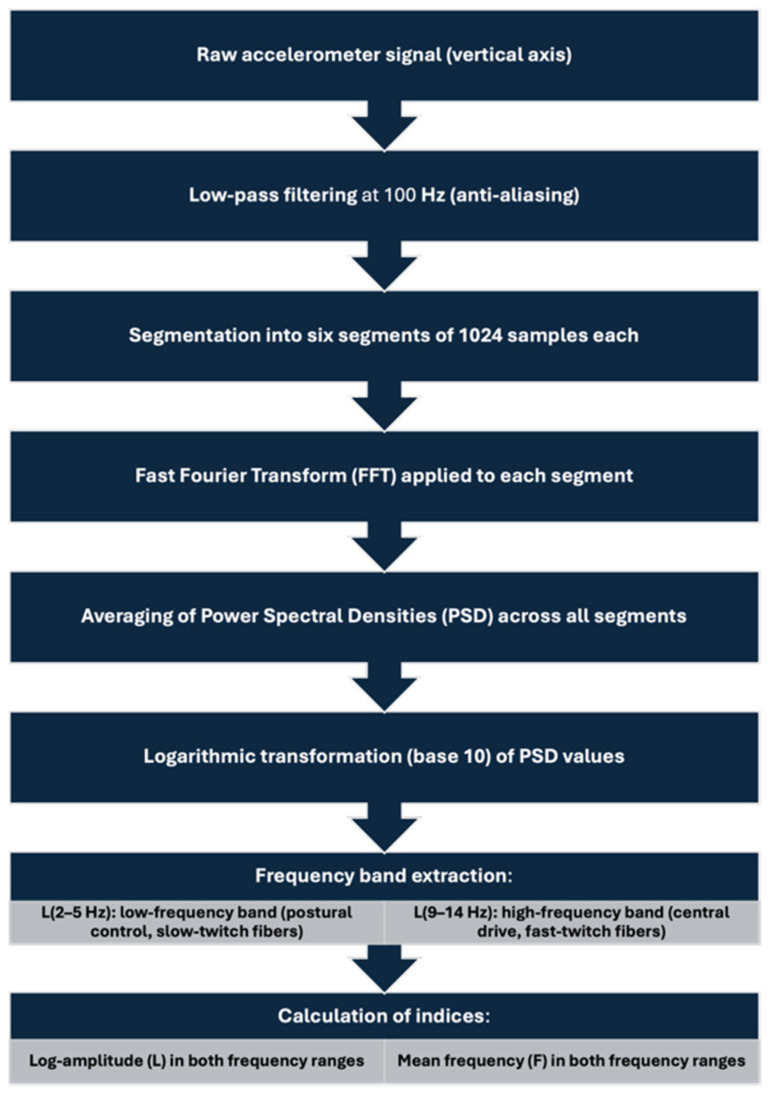
Signal processing diagram for tremor analysis.

The signal processing pipeline used to extract tremor characteristics from the raw accelerometer data is summarized in Figure 2. This step-by-step procedure includes filtering, segmentation, frequency-domain transformation, and the calculation of tremor-related indices based on predefined spectral bands.

The figure illustrates the full pipeline used to process accelerometer data collected from the vertical axis. After initial filtering and segmentation, each signal segment underwent fast Fourier transform (FFT) to obtain power spectral densities (PSD), which were averaged and log-transformed. Frequency band extraction was performed for two physiologically relevant ranges: 2–5 Hz (associated with postural control and slow-twitch fibers) and 9–14 Hz (associated with central drive and fast-twitch fibers). From these, tremor indices, log-amplitude (L) and mean frequency (F), were calculated.

The selection of low-frequency tremor L(2_5) and high-frequency tremor L(9_14) was based on prior studies investigating neuromuscular tremor and muscle fatigue in lower limbs, specifically related to motor unit recruitment and fatigue mechanisms [24]. Low-frequency tremor, L(2_5) Hz, is associated with postural control and voluntary motor control mechanisms and is linked to slow-twitch (Type I) muscle fiber recruitment, which is more active in sustained endurance activity [34]. High-frequency tremor, L(9_14) Hz, is related to central motor drive and fast-twitch (Type II) muscle fiber activation. Previous studies indicate that tremor in this range increases after high-intensity, fast movements, such as those observed in sprint training [23,35]. Previous upper limb tremor studies have extensively analyzed frequencies in the 8–12 Hz range. However, lower limb tremor differs due to the larger muscle mass and different motor unit recruitment strategies [33]. This study adapted frequency ranges that have been shown to correspond with neuromuscular function in weight-bearing muscles [24]. The chosen frequencies, L(2_5) and L(9_14), align with those used in previous research, particularly in studies on muscle fatigue and motor control post-exercise [36]. Although this method does not constitute a universally recognized gold standard, it has gained broad acceptance in neuromuscular research due to its non-invasive nature, methodological simplicity, and proven sensitivity to fatigue-induced alterations in muscle function [33,36]. Consequently, the selected frequency bands and analytical approach were deemed suitable for characterizing tremor phenomena associated with postural muscle control and central fatigue mechanisms in the lower limbs.

### 2.4. Statistical Analysis

Statistical analysis was performed using Statistica 13.0 and Microsoft Excel. The normality of the distributions of the variables describing tremor spectra was determined using the Shapiro–Wilk test (criterion *p* > 0.05). To examine the differences in the mean changes of tremor amplitude and frequency across conditions (resting tremor, sprint, and endurance training) relative to resting tremor, a repeated analysis of variance (ANOVA) was conducted. Two repeated factors were analyzed: condition (at rest, after endurance training, and after sprint training) and side (left limb and right limb). Levene’s test was used to assess the homogeneity of variance within the samples, and the Tukey test was applied for post hoc comparisons. Effect sizes were calculated using partial eta squared (η_p_^2^) and interpretated based on Cohen’s guidelines: η_p_^2^ ≥ 0.01 was considered a small effect, η_p_^2^ ≥ 0.06 a medium effect, and η_p_^2^ ≥ 0.14 a large effect. Additionally, an independent samples Student’s *t*-test was used to assess differences in tremor characteristics between the non-athlete group and the speed skater group. The magnitude of the effect was determined using Cohen’s *d*, where *d* = 0.2 was considered a small effect, *d* = 0.5 a medium effect, and *d* = 0.8 a large effect. The level of significance was set at α = 0.05.

## 3. Results

The ANOVA showed that condition (tremor at rest, after endurance training, and after sprint training) had a significant effect on the log-power index L(2_5) (F_1,18_ = 38.42; *p* < 0.0001; η_p_^2^ = 0.68). However, it did not indicate a significant effect of side, meaning that the tremor was not stronger in one limb compared to the other (F_1,36_ = 1.30; *p* = 0.27; η_p_^2^ = 0.07). Post hoc analysis showed that resting tremor was significantly different from tremor after both endurance training (*p* < 0.001) and sprint training (*p* < 0.001). However, there was no significant difference in tremor between endurance and sprint training conditions (*p* = 0.1014). Table 1 provides a summary of the mean (±SD) values of tremor amplitude (L) and frequency (F) indices across different conditions and limbs.

ANOVA revealed a significant effect of condition (tremor at rest tremor, after endurance training, and after sprint training) on the L(9_14) index (F_1,36_ = 19.19; *p* < 0.0001; η_p_^2^ = 0.51). This means that the changes in tremor amplitude depended on the specific condition being tested. The post hoc Tukey test showed that the tremor amplitude for the L(9_14) index was significantly different between rest and sprint training (*p* < 0.0001) and between rest and endurance training (*p* = 0.0002). This confirms that the tremor amplitude changes depending on the type of activity. Similar to what was observed in the lower frequency ranges L(2_5), no significant difference was found in the L(9_14) index between left and right limbs, which shows that the training program did not cause one limb to tremble more than the other (F_1,36_ = 2.85; *p* = 0.07; η_p_^2^ = 0.14). For the F(2_5) frequency, the ANOVA indicated a significant effect of training condition (F_1,18_ = 7.23; *p* = 0.0023; η_p_^2^ = 0.29). Similarly, for the F(9_14) index, training condition also had a significant effect (F_1,18_ = 6.61; *p* = 0.0036; η_p_^2^ = 0.27). However, in both cases (F(2_5) and F(9_14)), there was no difference between limbs. Post hoc analysis showed that F(2_5) during sprint training was significantly higher from resting tremor (*p* = 0.0079), and that the tremor increased significantly after endurance training (*p* = 0.0051). For the F(9_14) index, an increase relative to the resting tremor was observed only after sprint training (*p* = 0.0028). Conversely, no significant changes were observed for endurance training (*p* = 0.0070).

**Figure 3:**
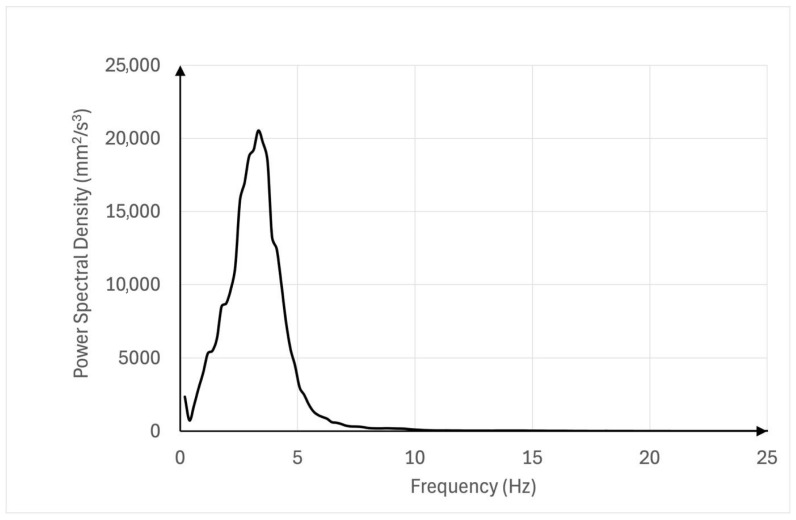
Power spectral density (PSD) plot of postural lower limb tremor in a representative participant. The plot illustrates the distribution of signal power across frequencies, with a dominant peak observed in the 4–6 Hz range. This indicates a major contribution of low-frequency components to the tremor signal, consistent with fatigue-related neuromuscular oscillations. The PSD was computed using log-transformed accelerometer data from the resting condition. This visualization provides an example of typical tremor characteristics observed across participants.

**Figure 4:**
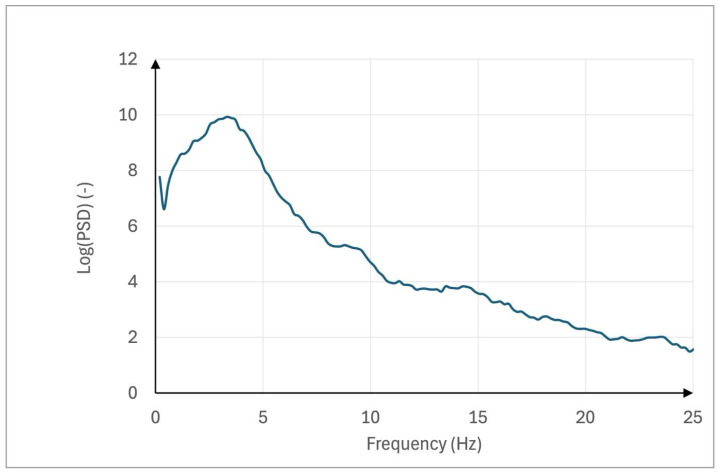
Power spectral density (PSD) plot on a logarithmic scale for a representative participant. The graph displays the distribution of signal power across frequencies, with the logarithmic scale highlighting subtle variations over a wide dynamic range. This representation enhances the visibility of both dominant and low-amplitude components, allowing a clearer interpretation of neuromuscular tremor patterns.

Figure 3 and Figure 4 illustrate examples of power spectral density (PSD) plots, showcasing the distribution of signal power across frequencies. In contrast, Figure 3 presents the PSD on a linear scale. 

The PSD on a logarithmic scale, emphasizing variations in power across a wider dynamic range while retaining the dominant frequency component, is presented in Figure 4. Together, these figures provide complementary perspectives on the signal’s frequency characteristics.

The findings demonstrate significant changes in tremor amplitude and frequency following both sprint and endurance training. Tremor amplitude in the low-frequency range (L(2_5) and high-frequency range (L(9_14)) increased significantly after both sprint and endurance training, indicating heightened neuromuscular fatigue compared to rest. Sprint training elicited a greater increase in high-frequency tremor amplitude (L(9_14) than endurance training, suggesting a distinct impact on neuromuscular function due to the activation of fast-twitch muscle fibers. Tremor frequency indices (F(2_5) and F(9_14)) also increased post-training, with sprint training having a stronger effect on high-frequency tremor compared to rest. Interestingly, no significant differences in tremor characteristics were observed between the left and right limbs, suggesting symmetrical neuromuscular adaptations, which may reflect the balanced technical and biomechanical demands of speed skating.

In order to establish comparative baseline values, measurements of resting tremor were also conducted among non-athletes. Below are the mean values of amplitude and frequency indices recorded for the right and left lower limbs in this group. For the left limb, the mean log-power amplitude in the 2–5 Hz range (L(2_5)) was 8.83 ± 0.46, and in the 9–14 Hz range (L(9_14)) it was 4.86 ± 1.02. The corresponding mean tremor frequencies were 3.18 ± 0.26 Hz for F(2_5) and 10.34 ± 0.28 Hz for F(9_14). In the right limb, the values were slightly lower in amplitude: L(2_5) averaged 8.20 ± 0.60, and L(9_14) 4.63 ± 0.72. The tremor frequencies in this limb were 3.01 ± 0.16 Hz for F(2_5) and 10.70 ± 0.14 Hz for F(9_14). These results indicate low variability in tremor characteristics across limbs in non-athletic individuals, with slightly higher tremor frequency observed in the right limb in the high-frequency band. The absence of training-specific neuromuscular fatigue likely explains the relatively symmetrical and moderate tremor values across both limbs.

**Figure 5:**
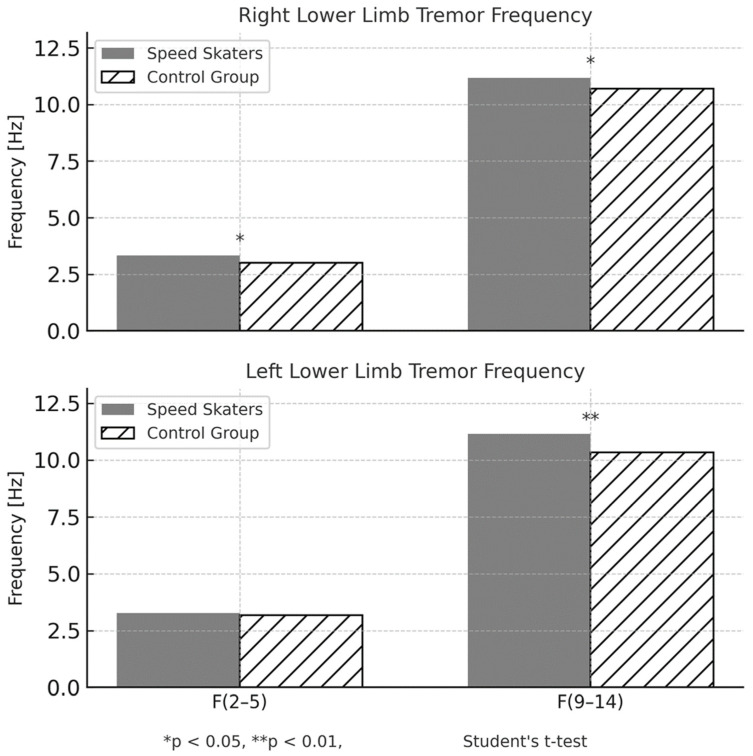
Tremor frequency indices in the 2–5 Hz (F(2–5)) and 9–14 Hz (F(9–14)) bands for the right and left lower limbs in speed skaters and control participants. Bars represent group means. Frequency values were significantly different between groups in several comparisons, as indicated by asterisks.

Significant between-group differences were observed in most tremor parameters. Group differences in tremor frequency parameters for both the low-frequency (F(2–5 Hz)) and high-frequency (F(9–14 Hz)) bands are illustrated in Figure 5.

Between-group differences in log-transformed tremor amplitude values for both frequency bands are presented in Figure 5.

**Figure 6:**
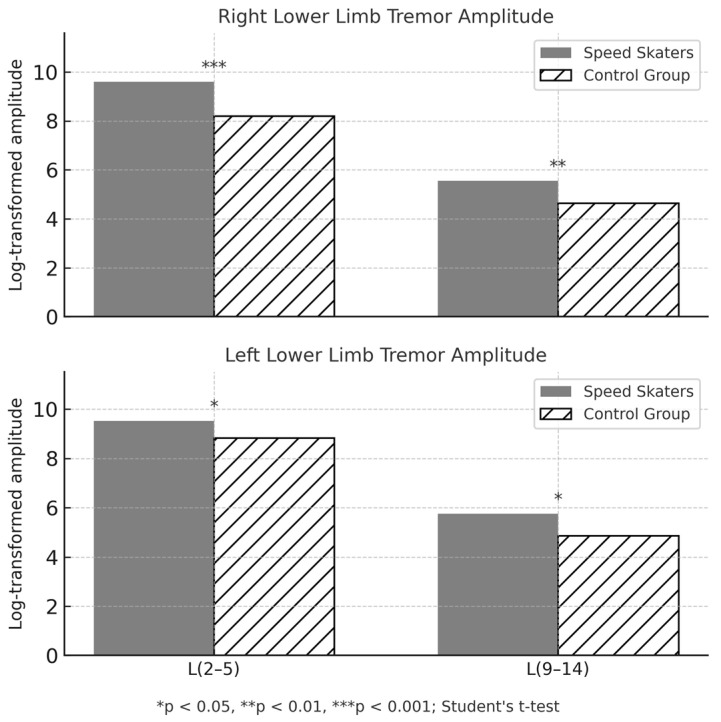
Log-transformed tremor amplitude (L) in the 2–5 Hz (L(2–5)) and 9–14 Hz (L(9–14)) bands for the right and left lower limbs in speed skaters and control participants. Bars represent group means. Significantly higher tremor amplitudes were observed in the speed skater group across both limbs and frequency ranges.

These results suggest that sprint training induces specific neuromuscular tremor responses in elite speed skaters that are not present in non-athletic individuals, including increased tremor amplitude and shifts in tremor frequency distribution. The differences are consistent across both limbs, reflecting sport-specific neuromuscular adaptations related to high-intensity training stimuli. Statistical analysis revealed no significant differences in resting tremor amplitude or frequency between the non-athletes and the speed skaters. Both groups demonstrated comparable values across low- and high-frequency tremor components (L(2_5)), (L(9_14)), (F(2_5)), and (F(9_14)), suggesting that sport-specific neuromuscular adaptations may become apparent only under post-exercise conditions. The demographic and anthropometric characteristics of both groups were generally comparable. Experimental and control groups revealed no significant differences in age (t = −1.13, *p* = 0.27, *d* = 0.37) or body height (t = −0.92, *p* = 0.36, *d* = 0.30). However, a statistically significant difference was observed in body mass, with control participants being heavier than elite speed skaters (t = −2.07, *p* = 0.046, *d* = 0.67). These findings indicate that physiological tremor at rest does not differ meaningfully between athletic and non-athletic populations, supporting the hypothesis that training-related differences emerge primarily in response to physical exertion. Figure 6 presents a comparison of log-transformed tremor amplitudes in two frequency bands (2–5 Hz and 9–14 Hz) for both lower limbs in speed skaters and control participants. The figure highlights significant differences in tremor characteristics between the groups.

## 4. Discussion

The findings of this study partially support the proposed hypotheses regarding the effects of sprint and endurance training on lower limb resting postural tremor. As hypothesized, significant differences in tremor characteristics were observed between the resting condition and both training conditions (sprint and endurance), as indicated by an increase in value in the L(2_5) and L(9_14) indices. These results suggest that both sprint and endurance training elicit neuromuscular fatigue, reflected in altered tremor amplitude. However, contrary to our hypothesis, no significant differences were observed in tremor amplitude between sprint and endurance training conditions for the L(2_5) index, indicating that the distinct physiological demands of these activities may not result in divergent tremor responses at rest. Additionally, the absence of significant differences between the right and left limbs confirms our hypothesis that the tremor response is symmetrical and not influenced by limb dominance or asymmetrical loading during training. This study is one of the first to investigate the specific characteristics of lower limb tremor in speed skaters, a population with unique neuromuscular demands due to the biomechanical and technical requirements of their sport. This approach enabled us to provide a more nuanced understanding of the tremor characteristics in the lower limbs of speed skaters, aligning both with the unique demands of their sport and with theoretical foundations in tremor research. By targeting these specific frequency ranges, our study bridges the gap between existing knowledge focused on upper limb tremors and the distinct neuromuscular patterns observed in lower limbs during high-performance activities. This perspective not only highlights the relevance of tailored frequency ranges but also underscores the importance of sport-specific analysis in neuromuscular research. The application of low-frequency ranges, such as L(2–5 Hz), and high-frequency ranges, such as L(9–14 Hz), in this study differs from those utilized in research conducted by Gajewski [33]. However, it is important to note that Gajewski’s studies primarily focused on investigating upper limb tremors. In contrast, the frequency ranges selected for the present study appear to be particularly effective in capturing the most pronounced changes in the tremor characteristics of the lower limbs. Moreover, the selection of low-frequency (e.g., L(2–5 Hz)) and high-frequency (e.g., L(9–14 Hz)) ranges in our study was informed by established literature and prior research in the field of tremor analysis. Low-frequency ranges (2–5 Hz) have been associated with voluntary muscle control and postural adjustments, as detailed in studies by McAuley [34] and Marsden [35], which explore physiological tremor characteristics under varying exertional conditions. On the other hand, high-frequency ranges (9–14 Hz) have been closely linked to central nervous system activity and have been extensively studied in the context of fatigue and neuromuscular performance [23]. The chosen frequency ranges enabled the simultaneous assessment of physiological and neuromuscular components of tremor, aligning with theoretical frameworks and prior findings in this field. Furthermore, this range selection facilitated the detailed examination of dynamic tremor frequency changes under various experimental conditions, including rest, sprint, and endurance activities, providing a more comprehensive understanding of the neuromuscular demands in speed skating.

In our study, we found no significant differences in tremor between the right and left limbs, supporting our hypothesis that the tremor response is symmetrical and not influenced by limb dominance or uneven loading during training. This contrasts with findings from Papale et al. [36] who reported that the dominant limb tends to show smaller tremor amplitudes compared to the non-dominant limb due to the better motor control and coordination developed from more frequent use in daily activities. Interestingly, our results suggest that training did not create a larger impact on one side over the other, and this symmetry may reflect balanced neuromuscular adaptations in both limbs. Additionally, despite initial assumptions that training might reveal differences related to thixotropy, which is the phenomenon where muscle stiffness decreases during and after activity and gradually returns during rest [37], no such differences were observed. However, our findings align with previous research that show exercise can lead to alterations in physiological tremor. For instance, Gajewski & Viitasalo [38] observed prolonged increases in tremor amplitude in response to intense muscular work, which demonstrated that sprint and endurance training significantly alter tremor amplitude and frequency. Nevertheless, unlike prior findings in asymmetrical sports, we did not detect side-specific effects in our participants. Similarly, Novak et al. [16] found increases in forearm tremor amplitude following maximal exercise in young swimmers. Another study focused on military training revealed that prolonged physical exertion and sleep deprivation during training altered the characteristics of physiological tremor. Intense physical activity may cause lasting changes in tremor amplitude, influencing psychomotor performance [30]. The current study extends these observations to the lower limb tremor of speed skaters, suggesting that both endurance and sprint training can elicit changes in tremor characteristics. This assertion is further substantiated by various scholars who assert that personalized training regimens can enhance postural stability and refine motor abilities, which are essential for the effective management of tremor. Such programs can be customized to address the distinct requirements of individuals, predicated on the specific attributes of their tremor [39]. The mechanisms underlying the observed changes in tremor remain unclear. The authors speculate that the increases in tremor amplitude and frequency may be related to central nervous system factors, such as alterations in motor unit recruitment and synchronization. Additionally, peripheral factors like metabolic changes and muscle fatigue may contribute to the tremor modifications [29,40,41]. While these studies provide insights into how training affects lower limb tremor, it is important to consider that the effects of training on tremor can vary based on the type, intensity, and duration of physical activity. Our results further demonstrate this variability, as significant changes in tremor characteristics were observed after both sprint and endurance training. Additionally, individual differences in neuromuscular control and adaptation to training may also play a significant role in tremor characteristics [42]. A notable limitation of this study is its relatively small sample size, which makes the findings potentially underpowered. It is possible that increasing the sample size by approximately 10 participants could have allowed for the detection of a side-specific effect, if present. Unfortunately, speed skating has a relatively low popularity, despite its status as an Olympic sport, and this posed significant challenges in recruiting a larger cohort of athletes. This limitation highlights the difficulties associated with conducting research in niche sports and underscores the need for collaborative efforts to expand participant pools in future studies. This study provides a foundational understanding of how speed skating training impacts muscle tremor, and several areas warrant further exploration. Examining tremor characteristics in athletes from other asymmetrical sports, such as cycling or tennis, could help elucidate the influence of sport-specific biomechanics on neuromuscular adaptations. Investigating how muscle tremor evolves over an athlete’s career could offer insights into the long-term neuromuscular adaptations associated with prolonged training. Future research should aim to uncover the underlying mechanisms driving changes in tremor characteristics, focusing on central nervous system adaptations. Furthermore, it would be valuable to employ multi-modal assessment techniques, such as electromyography (EMG) or biochemical fatigue markers, to enhance the physiological interpretation of tremor characteristics. Combining accelerometry with these complementary methods may offer a more comprehensive evaluation of both central and peripheral fatigue mechanisms. This assessment could provide a more comprehensive understanding of the physiological adaptations underlying the observed alterations in tremor. The merit of the employed research methodology is indisputably its non-invasive nature, which necessitates merely elementary technical training for the operation of the apparatus. This represents a relatively innovative strategy for examining the physiological behavior of the body and its reactions to the physical exertions performed. This study contributes to the growing body of research on physiological tremor by establishing its responsiveness to specific training modalities in speed skaters. A critical question that emerges is whether the tremor characteristics identified in this cohort are unique to speed skaters or representative of broader athletic populations. The distinctive biomechanical profile of speed skating characterized by sustained isometric contractions, symmetrical loading, and specific postural constraints may underlie the symmetry and spectral specificity of the tremor responses observed. In contrast to asymmetrical sports or general, non-athletic populations, these results may reflect a sport-specific pattern of neuromuscular adaptation [43,44]. To examine the potential specificity of these findings, it is instructive to consider analogous investigations. Gajewski et al. [33] analyzed forearm tremor in a large sample of youth athletes across multiple disciplines. Their results revealed a consistent spectral peak within the 8–12 Hz “physiological” range and significant sex-based differences in tremor amplitude. Interestingly, such sex-related variability was not observed in the current study, potentially reflecting both the homogeneity of the speed skater sample and differences in measurement site (upper vs. lower limb), as well as sport-specific neuromuscular control strategies. Similarly, Gajewski et al. [41] reported an increase in forearm tremor amplitude following maximal exertion in swimmers, a population engaged in rhythmic, bilateral movements analogous to those in speed skating. However, their study did not examine inter-limb differences, leaving open the question of whether the bilateral symmetry observed in our cohort is a hallmark of skating-specific neuromuscular adaptation. Conversely, investigations involving non-athletic individuals offer a contrasting perspective. Research on essential tremor [45,46] frequently reports asymmetrical tremor manifestation, heightened inter-individual variability, and a lack of association with physical exertion. These studies seldom explore the dynamic modulation of tremor in response to training, thus limiting direct comparability. Nonetheless, their findings underscore the potential role of sport-specific demands in shaping tremor characteristics.

In summary, while an exercise-induced increase in tremor amplitude appears to be a broadly generalizable phenomenon, the distinct frequency profiles, bilateral symmetry, and responsiveness observed in speed skaters likely reflect unique neuromuscular adaptations. Further comparative research including athletes from symmetrical and asymmetrical sports, as well as sedentary controls, is warranted to elucidate the underlying mechanisms and potential diagnostic applications of sport-specific tremor patterns [47]. Future research should aim to refine tremor measurement techniques and integrate them into broader athlete monitoring systems. 

This study has several limitations that should be addressed in future research. The study involved 19 speed skaters, which may limit the generalizability of the findings to larger and more diverse populations. The sample size can also reduce the statistical power, potentially obscuring subtle effects, such as side-specific differences in tremor characteristics. Consequently, future research should aim to include a more diverse and larger population of speed skaters to enhance the statistical power and generalizability of the results. Expanding the participant pool not only increases the robustness of the findings but also enables researchers to explore potential variations in performance and injury rates across different demographics within the sport. This approach could lead to more comprehensive insights into the factors influencing performance and health outcomes, ultimately contributing to improved training protocols and injury prevention strategies tailored for speed skaters. However, other research suggests that neuromuscular asymmetries do exist in elite athletes, particularly in unilateral sports like cycling or tennis [21]. Expanding the study to 30+ athletes would allow for greater differentiation between limb adaptations. A larger sample would also enable subgroup analysis (e.g., dominant vs. non-dominant limb), potentially revealing hidden asymmetries. The relatively small sample size, although reflective of the elite nature of the study population, may have reduced the statistical power of the secondary analyses, particularly those examining inter-limb differences. As a result, subtle asymmetries between limbs might not have been detected, even if present.

The participants were elite, well-trained speed skaters, which narrows the applicability of the results to other athletic populations or age groups. Differences in training backgrounds, neuromuscular development, or muscle composition might influence the observed tremor characteristics. The study controlled for factors like training abstinence and caffeine intake before measurements. However, other potential influences, such as psychological stress, hydration status, or nutritional intake should also be taken into account in future studies.

## 5. Conclusions

In conclusion, this study reinforces the value of physiological tremor analysis as a practical tool for assessing the neuromuscular impacts of sport-specific training. Our findings demonstrate significant changes in tremor characteristics following both sprint and endurance training, emphasizing the relevance of tremor analysis in understanding the physiological demands of these activities. The physiological tremor response also highlights the effectiveness of balanced training regimens in promoting uniform neuromuscular adaptations. Although no differences in resting tremor were observed between groups, post-exercise comparisons revealed that speed skaters exhibited significantly higher tremor amplitudes and altered frequency patterns compared to non-athletes. These findings suggest that sport-specific neuromuscular adaptations in elite athletes become apparent primarily under conditions of physical exertion. Expanding this research to diverse athletic populations and training regimens could further validate the use of tremor analysis as a non-invasive tool for monitoring neuromuscular health and fatigue.

## Figures and Tables

**Table 1:** Means (±SD) of variables describing tremor log-power (logarithmic amplitude indicators (L) of right and left lower limbs of speed skaters) and mean tremor frequencies (F) in selected frequency ranges of right and left limbs of participants (*n* = 19).

	Resting Tremor		After Endurance Training		After Sprint Ttraining	
Variable	Left Limb	Right Limb	Left Limb	Right Limb	Left Limb	Right Limb
L(2_5)	8.89 ± 0.89	8.60 ± 1.03	9.49 ± 0.86	9.47 ± 0.91	9.52 ± 1.00	9.60 ± 0.87
L(9_14)	4.86 ± 1.02	4.40 ± 0.99	5.38 ± 1.01	5.86 ± 1.61	5.75 ± 1.50	5.55 ± 1.07
F(2_5) [Hz]	3.19 ± 0.16	3.19 ± 0.16	3.28 ± 0.15	3.33 ± 0.23	3.26 ± 0.19	3.33 ± 0.23
F(9_14) [Hz]	11.34 ± 0.28	11.39 ± 0.31	11.22 ± 0.38	11.25 ± 0.41	11.15 ± 0.28	11.18 ± 0.33

## Data Availability

The datasets used and analyzed during the current study are available from the corresponding author upon reasonable request.
